# Editorial: Early diagnoses and treatments of uncommon breast cancers

**DOI:** 10.3389/fendo.2022.1042226

**Published:** 2022-10-18

**Authors:** Ernestina Marianna De Francesco, Veronica Vella

**Affiliations:** Endocrinology, Department of Clinical and Experimental Medicine, University of Catania, Garibaldi-Nesima Hospital, Catania, Italy

**Keywords:** breast cancer, uncommon breast cancer, hormone receptor, triple-negative breast cancer, epidemiology, metastasis, recurrence, prognosis

Breast cancer (BC) is the most frequently diagnosed malignant disease in women and the second cause of cancer-related death globally (1). Despite the enormous progress of the last 30 years, which allowed to improve considerably the clinical management of BC patients, mortality at 5 years after first diagnosis still affects nearly 13% of women. In the vast majority of cases, metastatic evolution is one of the main most important factors implicated in mortality; in addition, disease relapse may be associated with higher mortality rates.

The categorization of BCs into three major molecular subtypes, based on the presence of the estrogen receptor (ER) and progesterone receptor (PR) (ER-positive BCs), epidermal growth factor 2 receptor (HER2-positive BCs), or the lack thereof (Triple Negative Breast cancers-TNBCs) has allowed to get an immediately reliable tool to predict prognostic profiles and delineate treatment strategies. However, additional research efforts need to be undertaken to better detect populations at risk, develop improved tools for early diagnosis and treatment, and identify efficient prognostic and predictive biomarkers, particularly for uncommon forms of BC.

This Research Topic includes 3 case reports and 9 original articles focusing on certain novel molecular, biological and clinical features, together with epidemiological aspects that might be helpful in the early diagnosis and treatment of uncommon forms of BC.

Although BC is usually less frequently diagnosed in younger women (< 40 years), the disease is more likely to present in a more aggressive and advanced form compared to older women (> 40 years). By analyzing the clinicopathological characteristics and survival data of a large cohort of young women with early stage BC, Liu et al. have developed a predictive nomogram to identify potential risk factors of cancer-specific survival, that would help the clinicians in decision-making processes. Similar efforts have been made by the study of Tang et al. in which through a novel nomogram based on several independent prognostic factors the authors highlight that both radiation and adjuvant chemotherapy are significantly associated with favorable long-term overall survival (OS) and cancer-specific survival (CSS) probability in elderly primary operable TNBC patients. Another way to effectively stratify the high-risk and low-risk BC patients has been advanced by Min et al. establishing an autophagy-related 4-gene signature as significantly associated with an early relapse. As lymph node negative BC can coexist with distant metastasis Min et al. proposed a novel nomogram to better stratify patients who are at high-risk for developing distant metastasis. Moreover, by using a multiple databases and bioinformatic tools Wu et al. found SPINT1/2 (serine proteases acting as HGF activator inhibitors) as potential prognostic biomarkers for patients with BC.

As the majority of patients with early ER-positive BC will not experience a recurrence when treated with 5 years of adjuvant endocrine therapy, identifying those patients that can safely be excluded from additional adjuvant chemotherapy and/or extended adjuvant endocrine therapy has been a high priority over recent years and has led to the development of several commercial multiparameter genomic tests, including the 21-gene recurrence score (RS). Chen et al. conducted a study to assess the impact of the 21-gene RS in early BC patients confirming that the result was due to the estrogen module regardless of age.

Enhanced invasive behavior is frequently observed in primary small cell breast carcinomas (SCBCs). Zhu et al. perform the largest population-based study of SCBC, to gather information on incidence, clinic-pathological features, and prognostic factors. The authors confirm that SCBC is an infrequent and aggressive neoplasm with characteristics of poor differentiation; in addition, chemotherapy, surgery and stage were identified as important predictors of disease-specific survival (DSS) and OS.

It should be mentioned that the molecular profiling of uncommon forms of BC may also have a prognostic significance and may lead the decision-making processes. This is the case for metaplastic BC, which represents a rare and aggressive form of BC with uncertain clinical outcome due to the lack of specific treatment options. In this regard, Hu et al. clarified the prognostic role of HER2 in metaplastic BC, by performing a propensity score-matched analysis in almost 3000 patients over a 28-months’ time. Data show that despite ER status and HER2 status have no impact on DSS, HER2 positive status and post-mastectomy radiotherapy are associated with better prognosis. Further dissecting the possible therapeutic options for metaplastic BC, another case report by Fu et al. shows that a 58 years old woman was unresponsive to standard adjuvant chemotherapy provided as first-line treatment, and chemotherapy combined with anti-angiogenic treatment administered as a second-line therapy. However, a partial response was achieved after treatment with immunotherapy (toripalimab) in combination with anti-angiogenic therapy (anlotinib). These findings highlight the need to gather more data on metaplastic BC for better stratifying and treating patients.

Gao et al. presented a case report of a peritoneal metastasis from an uncommon invasive lobular carcinoma (ILC) of the breast with resistance to therapy due to acquired ESR1 and PI3KCA mutations revealed through whole exome sequencing (WES). For this reason, the authors propose WSE as a supplementary technique for early diagnosis of metastatic BC patients.

Rarely BCs can appear as phyllodes tumors giving rise to hypoglycemia due to the production of tumor-derived high molecular weight form of insulin like growth factor 2 (IGF-2). This is the case reported by Zhao et al. in which the surgical resection of the tumor successfully resolved the hypoglycemia associated symptoms.

For improving early diagnosis of invasive BCs, Zhao et al. developed a clinical prediction tool using molecular classification, tumor size and Cooper’s ligament status to predict the probability to have an axillary lymph node tumor burden in addition to the sentinel lymph node biopsy.

As a result of ongoing breakthroughs in prognostic and predictive biomarkers as well as in cancer therapy, cancer patients’ survival rates have grown considerably. However, further efforts may help to convert BC research into clinical practice in the future for early detection and improvement of cancer survival. Moreover, new biomarkers are warranted to individualize treatment. This will provide health professionals with a powerful decision-making tool that can be used to better manage BC patients.

## Author contributions

All authors listed have made a substantial, direct, and intellectual contribution to the work and approved it for publication.

## Funding

EF was supported by Fondazione AIRC (Start-Up Grant 21651).

## Conflict of interest

The authors declare that the research was conducted in the absence of any commercial or financial relationships that could be considered as a potential conflict of interest.

## Publisher’s note

All claims expressed in this article are solely those of the authors and do not necessarily represent those of their affiliated organizations, or those of the publisher, the editors and the reviewers. Any product that may be evaluated in this article, or claim that may be made by its manufacturer, is not guaranteed or endorsed by the publisher.
